# MedFuse: dual-stream fusion of convolutional and vision transformer-based features for enhanced medical image classification

**DOI:** 10.3389/fmed.2026.1901033

**Published:** 2026-09-29

**Authors:** Yajing Ren, Ling Hai, Zheng Gu, Wen Liu

**Affiliations:** Artificial Intelligence and Smart Mine Engineering Technology Center, Xinjiang Institute of Engineering, Urumqi, China

**Keywords:** convolutional neural networks, dual-stream architecture, feature fusion, medical image classification, vision transformers

## Abstract

**Introduction:**

Medical image classification is fundamental to computer-aided diagnosis. Limited labeled samples, subtle inter-class differences, and heterogeneous lesion morphology make it difficult for a single representation to capture all relevant cues. This study investigates MedFuse, a simple dual-stream framework that combines convolutional neural network (CNN) features with representations from a pretrained vision foundation model (VFM).

**Methods:**

The CNN branch is optimized on the target dataset to emphasize task-adaptive local texture and morphology, whereas the VFM branch is kept frozen to provide a stable global semantic representation. The two pooled feature vectors are concatenated and passed to a common classification head. Accordingly, MedFuse is positioned as a systematic and reproducible fusion baseline. The framework is evaluated across all 12 two-dimensional MedMNISTV2 subsets using multiple CNN-VFM backbone pairs and is compared with alternative weight- and attention-based fusion schemes.

**Results:**

Simple late feature concatenation was competitive with, and often more stable than, more parameterized fusion modules across the evaluated settings. Moreover, increasing the size of the VFM backbone did not consistently improve classification performance.

**Discussion:**

These findings demonstrate that frozen foundation-model representations can complement trainable convolutional features in small-scale medical image classification, while also showing that increased fusion complexity or larger VFM backbones does not necessarily translate into better performance. MedFuse therefore provides a simple and reproducible baseline for systematically studying CNN-VFM feature complementarity in medical image classification.

## 1 Introduction

Medical image classification serves as a core component in computer-aided diagnosis, which helps clinicians automatically and accurately recognize lesion patterns from diverse imaging modalities such as x-rays, CT scans, and pathological slices (1). Convolutional Neural Networks (CNNs) have long been the mainstream method for medical image analysis, owing to their outstanding performance on large-scale natural image datasets such as ImagNet (2). However, medical imaging proposes unique challenges in practical applications. These datasets generally require high-cost manual annotation, suffer from limited data volume, and contain subtle inter-class distinctions as well as obvious intra-class variations (3). Such inherent characteristics frequently restrict the performance of conventional CNN models, resulting in weak generalization capability and insufficient sensitivity to subtle pathological changes in clinical scenarios (4).

To address the scarcity of annotated medical data, most existing studies adopt a transfer learning strategy: models are first pre-trained on massive natural image datasets and then fine-tuned on medical imaging data for downstream tasks (5). Although this approach improves model performance to a certain extent, it still has an inherent performance upper bound caused by the domain semantic gap. Feature knowledge learned from natural scenes cannot fully match the pathological features required for medical diagnosis, making generic pre-trained models less adaptable to clinical classification tasks (6). With the emergence of Vision Transformers, visual feature learning has entered a new stage. The self-attention mechanism enables these models to effectively capture long-range global contextual dependencies (7). As a typical representative, VFM deliver richer semantic information and stronger generalization ability when transferred to medical tasks, and can better fit structural features in medical images (8). In fact, CNNs and VFMs have their own unique strengths in medical image classification. Small and medium-sized CNN models excel at extracting fine-grained local textures and maintaining spatial invariance, which is critical for identifying tiny morphological and textural lesion features (9). In contrast, VFMs are good at modeling global context and high-level semantic correlations, showing obvious advantages in analyzing organ morphology, lesion distribution, and the spatial relationships between pathological areas and surrounding normal tissues (10). Unfortunately, most current studies still focus on optimizing single-branch models alone and fail to fully to exploit the complementary strengths of CNNs and VFMs.

Clinical diagnosis typically relies on physicians’ dual perspectives: observing local lesion details while integrating overall contextual information to make comprehensive and accurate judgements (11). To balance fine-grained local analysis and global contextual perception in model deign, some studies adopt knowledge distillation techniques, which transfer effective knowledge from large teacher models to lightweight student models. Rather than completely replicating the full capability of large standalone models, these methods aim to achieve a better balance among model performance, inference efficiency and generalization performance (12). Other related multimodal fusion research mainly focuses on fusing data collected from different medical imaging devices (13). Despite these efforts, most existing methods are developed and validated on a single type of medical imaging dataset or a limited number of disease-specific datasets, and their performance across diverse imaging modalities, anatomical regions, and classification tasks remains insufficiently investigated.

To address these limitations, we study MedFuse as a general dual-stream CNN-VFM framework for medical image classification. The framework deliberately uses a simple late-fusion design: a task-trained CNN provides local and morphology-sensitive features, while a pretrained VFM with frozen parameters supplies stable global semantic features; the pooled feature vectors are concatenated before classification. The contribution is the systematic investigation of this simple design across 12 MedMNISTV2 tasks, multiple heterogeneous CNN-VFM backbone combinations, VFM scales, and competing fusion strategies. The study therefore focuses on empirical evidence about complementarity, stability, and backbone compatibility. The main contributions of this study are:

1. A unified dual-stream evaluation framework that combines a trainable CNN branch with a frozen pretrained VFM branch and keeps the fusion operation intentionally simple and reproducible.
2. A broad benchmark across all 12 two-dimensional MedMNISTV2 subsets, covering pathology, x-ray, OCT, ultrasound, dermoscopy, microscopy, fundus, and CT-derived tasks.
3. A systematic analysis of VFM scale, CNN-VFM backbone pairing, and three feature-fusion strategies (concatenation, adaptive weighting, and attention), showing that increased model or fusion complexity does not guarantee better medical-image classification performance.
4. An empirical characterization of the practical strengths and limitations of late concatenation, including information preservation, low fusion-layer complexity, increased feature dimensionality, and limited explicit modeling of cross-branch interactions.

## 2 Related work

### 2.1 Applications of CNNs in medical image analysis

CNN-based learning approaches are widely adopted to mitigate the shortage of high-quality annotated data in medical image analysis. For instance, Frees et al. (14) systematically compared the pre-training performance of RadImageNet and ImageNet on breast cancer and ACL tear classification tasks. Their experiments outcomes indicated that promising results depended on specific network architecture combinations, yet they found no solid evidence proving the consistent superiority of RadImageNet pre-training. By comparison, a multi-center study on bone tumor classification drew more definite conclusions, confirming that RadImageNet-pre-trained models delivered much better performance on external test sets than ImageNet-pre-trained counterparts (15). Such inconsistent findings highlight how the modality diversity of pre-training data correlates closely with the performance of downstream medical tasks. The RadiologyNET consortium also pointed out that severe domain mismatch between pre-training data and downstream medical samples can greatly weaken the effectiveness of pre-trained parameters, especially when the target dataset has extremely limited annotations (16). Beyond data-driven optimization, many researchers turn to lightweight CNN design to accommodate the limited computing resources and real-time deployment demands of clinical scenarios. For example, the Lite-V2 architecture achieves reliable classification accuracy and generalization in multi-class lung pathological image recognition while keeping computational overhead low (17). Even so, CNNs still have inherent weaknesses. Although they excel at extracting local image features, their constrained receptive fields hinder the capture of long-range dependencies and overall global contextual information.

### 2.2 Vision transformer-based visual foundation models

Vision Transformer (ViT) architectures rely on self-attention mechanisms to implement global feature modeling, allowing models to capture global semantic information and structural relationships inside medical images. Recent research has verified that VFMs, including AIMv2, DINOv2, and SAM2, perform well on various medical classification tasks, ranging from mammography screening to skin lesion diagnosis (18). To further improve the classification accuracy of medical images, many studies have developed domain-specific VFMs tailored for clinical scenarios. As a representative work, the SPECTRE model adopts self-distillation and vision-language alignment strategies, achieving strong performance in both zero-shot and fine-tuning settings across multiple CT analysis tasks (19). In addition, several studies integrate visual encoders with large language models to build multimodal medical learning frameworks. Equipped with anatomical prior guidance, these lightweight vision-language models outperform traditional vision-only models in multiple tasks, such as OCT image classification (20). Further comparative studies show that pure visual models may achieve competitive results under ideal experimental conditions, while vision-language models can exhibit better robustness when processing low-quality images with distortion or uneven imaging quality (21). Despite this progress, how to combine the local inductive biases of CNNs with the global representations of Transformer-based models in a simple, reproducible, and broadly evaluated medical-image classification setting remains an open empirical question. In this study, we therefore focus on systematically evaluating late CNN-VFM feature concatenation.

### 2.3 Multi-Model and feature fusion methods

Multi-model and feature fusion techniques have proven effective in boosting the robustness and overall performance of deep learning models. With the rapid development of deep learning technologies, feature-level fusion has gradually become the mainstream optimization scheme in visual tasks. Dual-stream network structures, in particular, are widely used to fuse spatiotemporal information and multimodal data for improved feature representation (22). In the field of medical image analysis, existing works have mainly focused on fusing heterogeneous imaging modalities or combining CNN-derived deep features with manually crafted features (23). In terms of ensemble learning, feature-enhanced stacking frameworks have achieved remarkable results in breast ultrasound classification by concatenating deep CNN features with predictive outputs from basic baseline models (24). For practical scenarios with limited computational resources, lightweight model designs have also been extensively explored. Relevant studies demonstrate that pure CNN ensembles methods can effectively reduce memory usage, while hybrid ensemble structures strike a better balance between classification accuracy and computational efficiency (25). Further studies suggest that combining heterogeneous architectures with calibration strategies is key to improving the robustness of medical image classification (26). Heterogeneous ensemble frameworks that integrate various ViT, CNN, and Multiple Instance Learning (MIL) models, together with tailored loss functions and calibration strategies, have substantially boosted cross-center generalization ability (27). For example, ConvTransGFusion uses a learnable dual-attention gating mechanism to fuse ConvNeXt and Swin Transformer, outperforming existing state-of-the-art methods on multiple medical imaging tasks (28). Recent hybrid CNN-Transformer architectures have demonstrated great potential in various medical image classification tasks. In lung CT diagnosis, the AACNN-ViT model combines CNN and Transformer modules through an adaptive attention fusion module. This hybrid design improves overall classification accuracy and greatly enhances the models’ capability to identify minority lesion samples (29). For breast x-ray BI-RADS grading tasks, a multi-scale ResNet-Transformer hybrid structure has been proposed. It leverages ResNet-50 to capture detailed local features and lightweight self-attention mechanisms to model long-range global future relationships. By utilizing a distance-aware loss function that accommodates the ordinal attributes of BI-RADS classification criteria, this method achieves reliable and high-quality diagnostic results (30). Similarly, MedViT and other hybrid models have been developed for chest x-ray multi-label classification. These methods combine domain-specific data augmentation, transfer learning strategies and self-attention modules. Such integrated designs allow the model to sustain high accuracy and strong robustness on complex disease datasets, while maintaining stable recognition performance for rare conditions including fibrosis (31).

Despite these advances, most existing models are tailored to only a limited set of diseases (Table 1). Accordingly, improving the generalization ability of such models remains an important direction for further research.

**Table 1:** Comparison with representative dual-stream/CNN-transformer fusion studies.

Reference	Dataset(s)	CNN/backbone	Transformer/VFM	Fusion strategy	Training strategy	External validation	Main contribution/limitation
Qezelbash-Chamak and Hicklin (28)	General image classification	ConvNeXt	Swin Transformer	Learnable dual-attention gating	Joint/hybrid training	Not emphasized	Learnable cross-branch fusion; not medical-domain focused.
Rahman and Rahman (29)	Lung CT	CNN	ViT	Adaptive attention fusion	Hybrid training	No	Disease-specific design; limited task breadth.
Ullah et al. (32)	Pantograph wear	ShuffleNetV2	DeiT-Tiny	Concat, weighted, bilinear, cross-attention, gated	Frozen dual backbones + trainable fusion	No	Systematic multi-fusion analysis and cross-validation; non-medical domain.
Ullah et al. (33)	Brain MRI, breast ultrasound, retinal fundus	Custom MobileNet + handcrafted features	—	Feature + decision-level fusion	Task-specific training	Across three datasets, not train-on-one/test-on-another	Explainable multi-dataset fusion; not a CNN-VFM dual-backbone architecture.

## 3 Methodology

### 3.1 Problem definition

The objective of MedFuse is to evaluate whether complementary CNN and VFM representations can be combined effectively through a simple late-fusion design. Formally, we define the medical image classification task on a dataset $D=\{(I_{s},y_{s}){\}}_{s=1}^{n}$, where *n* denotes the number of annotated samples, $I_{s}\in R^{H\times W\times 3}$ represents a 2D medical image, and $y_{s}$ is the corresponding ground-truth label. MedFuse processes each input image through two parallel feature-extraction streams. The local stream uses a CNN backbone to learn task-adaptive texture, edge, and morphological representations from the target dataset, whereas the global stream uses a pretrained Transformer-based VFM to provide a stable semantic representation. In the implementation, the VFM backbone is completely frozen throughout training. After branch-wise global pooling, the two feature vectors are directly concatenated and passed to a trainable classification head; no learned weighting, gating, adaptive fusion, or cross-attention is used in the Fusion Module. Accordingly, the methodological contribution of MedFuse is a systematic and reproducible evaluation framework for studying the complementarity, backbone compatibility, scale sensitivity, and practical trade-offs of simple CNN-VFM late fusion across diverse medical image classification tasks. During optimization, only the CNN branch and the downstream fusion and classification layers are updated, while the VFM parameters remain fixed.

### 3.2 Method model

As illustrated in Figure 1, MedFuse contains two parallel feature extractors, branch-wise global pooling, late feature concatenation, and a shared classification head. The default configuration used in the main comparison pairs ResNet-18 with DINOv2-ViT-S/14. ResNet-18 yields a 512-dimensional pooled feature vector, and DINOv2-ViT-S/14 yields a 384-dimensional representation, producing an 896-dimensional fused vector before the classifier. Other evaluated backbones retain their native pooled embedding sizes: ResNet-50 (2048), DenseNet-121 (1024), ConvNeXt-Tiny (768), DINOv2-ViT-B/14 (768), DINOv2-ViT-L/14 (1024), BEiTv2 (768), and Swin-T (768). Thus, the input width of the classifier changes with the selected backbone pair, whereas the same head design is used and only the final output dimension is adapted to the number of classes/labels in each dataset. An overview of the evaluated architecture is presented in Figure 2, where the complementary local and global features extracted from CNN and VFM branches are fused before classification.

**Figure 1:**
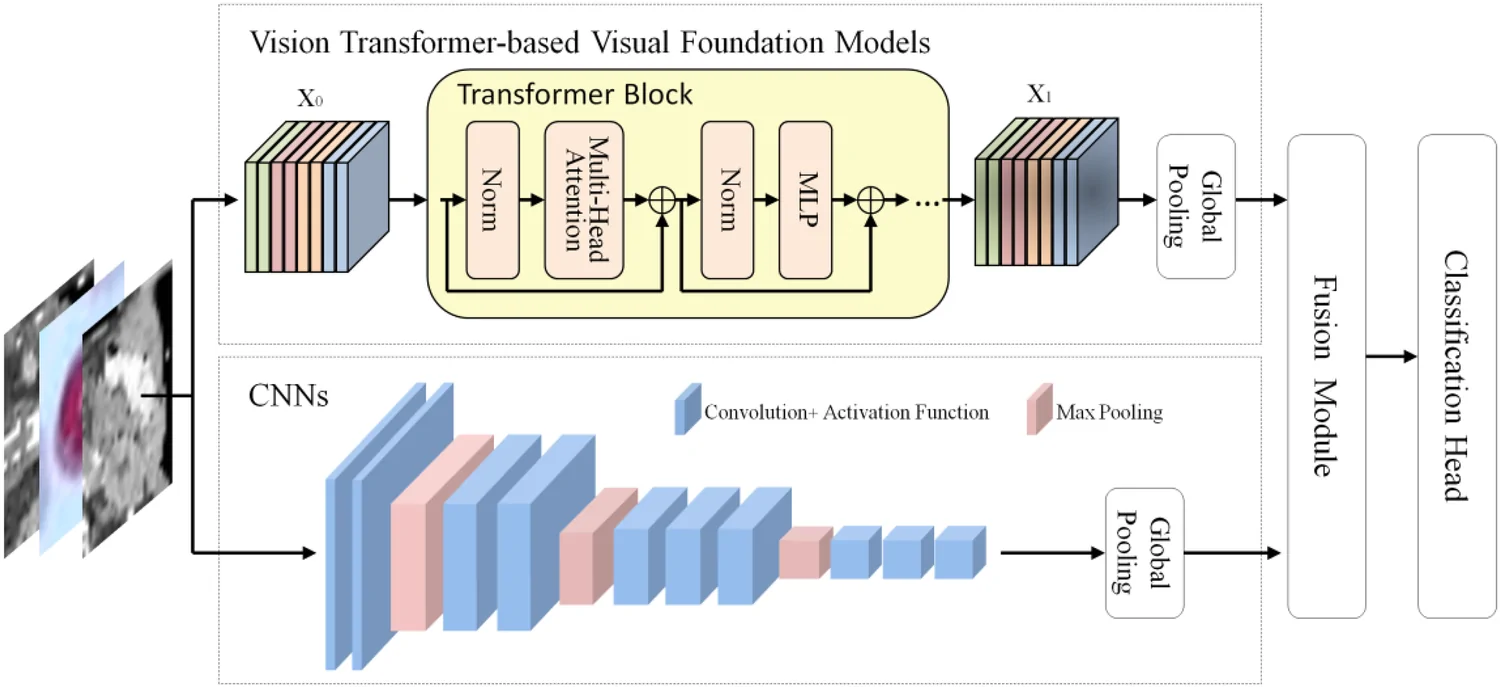
Architecture of the medical image classification model based on dual-stream fusion of VFMs and CNNs.

**Figure 2:**
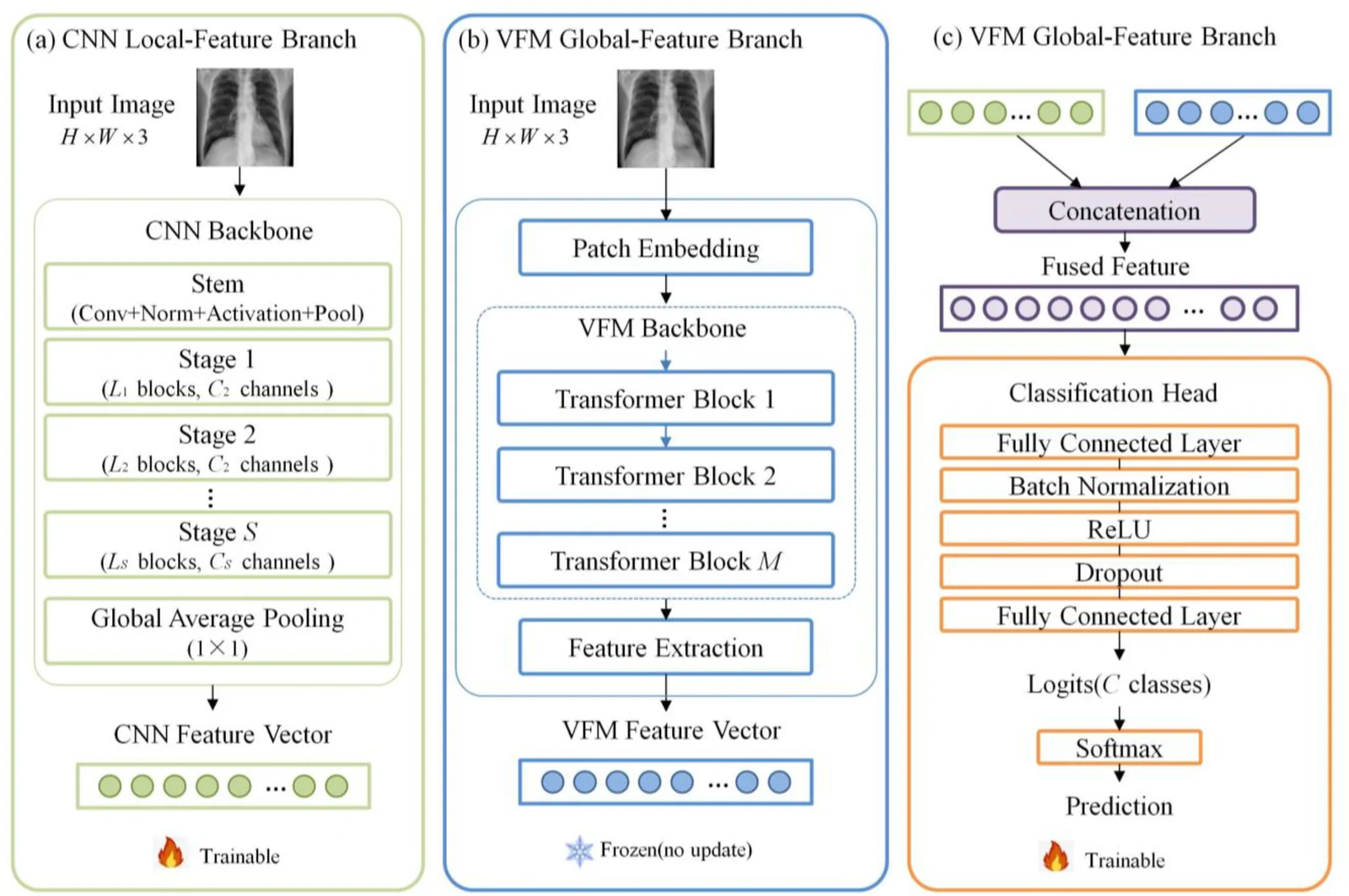
Overview of the evaluated CNN–VFM late-fusion framework. The network consists of three components: **(a)** a trainable CNN local-feature branch for extracting fine-grained spatial representations, **(b)** a frozen VFM global-feature branch for capturing high-level semantic features, and **(c)** direct feature concatenation followed by a trainable classification head for final prediction.

In the dual-stream feature extraction module, the local feature stream adopts a CNN backbone to extract local details, textures, and spatial structural information from the input images. The CNN gradually extracts hierarchical features through a series of convolutional layers, pooling layers, and nonlinear activation functions. The operation of the *L* -th convolutional layer can be formulated as:

$$F_{\left(i\right)}=\sigma (\mathrm{Con}v_{r\times r}(F_{\left(i-1\right)})+b_{\left(i\right)}),i=1,2\cdots L$$ (1)

Where, $F_{\left(i-1\right)}\in R^{h_{i-1}\times w_{i-1}\times c_{i-1}}$ denotes output feature map of the i−1 -th layer, $b_{i}\in R^{c_{l}}$ is the bias vector for the *i* -th layer, $\mathrm{Con}v_{r\times r}$ denotes the convolution operation with a kernel size of r×r, and σ(⋅) represents the nonlinear activation function. After being processed by *L* convolutional layers, the final extracted features can be expressed as:

$$F_{CNN}=f_{CNN}(I;\theta_{CNN})\in R^{h\times w\times c}$$ (2)

Where, *I* denotes the input image and $\theta_{CNN}$ represents all learnable parameters of the CNN.

The global feature stream utilizes a VFM to capture high-level semantic and contextual information from images. Initially, the model splits the input image *I* into *N* fixed-size patches:

$$P=[p_{1},p_{2},\cdots ,p_{N}]\in R^{N\times (P_{h}\times P_{w}\times C_{in})}$$ (3)

Each patch is then mapped to initial vector representation via linear projection:

$$z_{t}=W_{p}\cdot \mathrm{flatten}(p_{t})+b_{p}\in R^{d_{\mathrm{model}}}$$ (4)

Where, flatten(⋅) projects the patch $p_{t}$ into a 1D vector, $W_{p}\in R^{d_{\mathrm{model}}\times (P_{h}\cdot P_{w}\cdot c)}$ is the projection weight matrix, $b_{p}$ is the projection bias vector, and $d_{\mathrm{model}}$ denotes the hidden dimension of the Transformer.

To preserve spatial positional information, learnable position embeddings $E_{\mathrm{pos}}\in R^{(N+1)\times d_{\mathrm{model}}}$ are introduced to form the final embedding:

$$Z=Z_{N}+E_{\mathrm{pos}}$$ (5)

Subsequently, *Z* is processed through *M* layers of the Transformer encoder:

$$F_{\mathrm{VFM}\_\mathrm{vit}}=\mathrm{TransformerBloc}k^{\left(M\right)}(Z)$$ (6)

Each Transformer block mainly consists of a Multi-Head Self-Attention (MHSA) module and a feed-forward network. The multi-head attention is computed as:

$$\mathrm{MultiHead}(Q,K,V)=W_{o}(\mathrm{Concat}(\mathrm{hea}d_{1},\cdots ,\mathrm{hea}d_{A}))$$ (7)

Where, $W_{o}$ denotes the output projection matrix. Each attention head is calculated as:

$$\mathrm{hea}d_{a}=\mathrm{softmax}(\frac{Q_{a}K_{a}^{T}}{\sqrt{d_{k}}})V_{a}$$ (8)

Where, $Q_{a}$, $K_{a}$, and $V_{a}$ are derived from the input sequence *Z* using learnable weight matrices $W_{q}^{\left(a\right)}\in R^{d\times d_{q}}$, $W_{k}^{\left(a\right)}\in R^{d\times d_{k}}$, and $W_{v}^{\left(a\right)}\in R^{d\times d_{v}}$, and the dimensions of $Q_{a}$,$K_{a}$, $V_{a}$ are denoted as $d_{q}$,$d_{k}$, $d_{v}$, respectively.

To limit extra parameters and avoid early interaction between heterogeneous feature maps, MedFuse performs late fusion after branch-wise global pooling. Let $F_{CNN}\in R^{d_{CNN}}$ and $F_{VFM}\in R^{d_{VFM}}$ denote the pooled feature vectors. The default fusion operation is direct concatenation, $F_{\;fused}=[F_{CNN};F_{VFM}]\in R^{\left(d_{CNN}+d_{VFM}\right)}$. No learned weighting, gating, or cross-attention is applied in the default Concat variant, and no dimensionality reduction is performed before concatenation. This choice preserves the complete feature coordinates from both branches and adds negligible parameters in the fusion operation itself.

$$F_{\mathrm{fused}}=\mathrm{concat}(F_{\mathrm{CNN}},F_{\mathrm{VFM}})$$ (9)

The classification head maps the fused features to the final classification logits $H^{\left(2\right)}$:

$$H^{\left(k\right)}=\Phi_{k}(F_{\mathrm{fused}})$$ (10)

Where, $\Phi_{k}(\cdot )$ denotes the *k* -th transformation block, defined as:

$$\Phi_{k}(x)=\mathrm{Dropou}t_{\rho_{k}}(\mathrm{ReLU}(\mathrm{BN}(W_{k}x+b_{k})))$$ (11)

​ Where, $\rho_{k}$ denotes the dropout rate of each transformation block. The model uses the cross-entropy loss as the optimization objective, which normalizes predictions into a probability distribution via the Softmax function and computes the error relative to the ground-truth labels y:

$$L_{CE}=-\log(\frac{\exp(W_{y}H^{\left(k\right)}+b_{y})}{\sum_{\;j=1}^{C}\exp(W_{j}H^{\left(k\right)}+b_{j})})$$ (12)

Where, *C* denotes the number of classes.

## 4 Experimental results

### 4.1 Dataset

This paper uses the 12 two-dimensional subsets of MedMNISTV2 as a standardized benchmark. The collection spans colon pathology, chest x-ray, dermoscopy, retinal OCT, breast ultrasound, blood-cell microscopy, kidney-cortex microscopy, fundus imaging, and abdominal CT-derived organ classification. MedMNISTV2 provides standardized train/validation/test splits and corresponding classification labels. The 2D tasks include multi-class, binary, multi-label, and ordinal-label settings; therefore, labels are used in the task-specific format distributed by MedMNIST rather than being newly annotated by the authors.

The 12 subsets differ substantially in modality, texture, anatomical scale, and label structure. PathMNIST and TissueMNIST emphasize microscopic texture; BloodMNIST emphasizes cell morphology; ChestMNIST and PneumoniaMNIST use radiographic patterns; OCTMNIST and RetinaMNIST contain retinal structure; BreastMNIST is ultrasound; DermaMNIST is dermoscopy; and OrganA/C/SMNIST are CT-derived organ slices.

### 4.2 Experimental setup

We adopted consistent preprocessing and core training settings across all experiments. All input images were resized to 224 × 224 pixels using bilinear interpolation and converted to three-channel inputs. For originally grayscale images, the single channel was replicated three times to form an RGB-format input. All models were optimized using the Adam optimizer with a learning rate of 0.001 and a batch size of 32. Early stopping was employed to mitigate overfitting. For the modified Swin-T baseline, the embedding dimension was set to 48, the stage depths to (2, 6), and the numbers of attention heads to (2, 4, 8, 16). The same classifier-head design was used across the compared models where applicable, with only the input feature dimension and task-specific output dimension adjusted according to the backbone and dataset.

The backbone architectures were selected to evaluate the applicability of MedFuse across different architectural families and model scales. Specifically, the CNN candidates included ResNet-18 and ResNet-50 as representative residual networks, DenseNet-121 as a densely connected architecture, and ConvNeXt-Tiny as a modern convolutional architecture. For the VFM branch, DINOv2 variants at different model scales were evaluated together with representative Transformer-based alternatives, including BEiTv2 and Swin-T. These combinations allow us to investigate whether the performance of MedFuse depends on a particular backbone architecture or model scale.

During MedFuse training, the pretrained VFM backbone was kept completely frozen, whereas the CNN branch and the subsequent fusion and classification layers were trainable. This asymmetric training strategy was adopted to preserve the transferable representations learned by the pretrained VFM while allowing the CNN branch and classifier to adapt to task-specific medical image characteristics. Freezing the VFM backbone also reduces the number of trainable parameters and the optimization cost, which is particularly beneficial for relatively small medical imaging datasets.

All experiments were conducted on a workstation equipped with an NVIDIA GeForce RTX 3090 GPU with 24 GB of memory. The models were implemented in Python using PyTorch 2.1.0 under a Linux operating system, with CUDA 11.8 for GPU acceleration. Unless otherwise specified, all experiments were conducted under the same software and hardware environment to ensure fair comparisons among different models and configurations. The evaluation metrics include accuracy (ACC) and area under the receiver operating characteristic curve (AUC).

### 4.3 Experimental results analysis

#### 4.3.1 Comparison of single model baselines and dual-stream fusion model performance

Table 2 compares MedFuse with single-model baselines across the 12 MedMNISTV2 subsets. All experiments were independently repeated three times, and the results are reported as the mean ± standard deviation across the three runs. The results show substantial task-dependent variation among CNN and Transformer architectures. The default MedFuse configuration is competitive on many subsets and attains the highest AUC in several tasks, particularly PathMNIST and multiple organ-related datasets. Because all experiments use the benchmark's internal train/validation/test splits, these results support cross-task robustness within MedMNISTV2 but should not be interpreted as external clinical generalization. Although MedFuse achieves consistent improvements on several datasets, the performance differences are relatively modest on some tasks. Therefore, these improvements should be interpreted with appropriate caution, particularly when the differences are comparable to the variability observed across the three independent runs.

**Table 2:** Performance comparison of single model baselines and dual-stream fusion model.

Methods	PathMNIST		ChestMNIST		PneumoniaMNIST		OCTMNIST	
	AUC	ACC	AUC	ACC	AUC	ACC	AUC	ACC
ResNet-18	0.950 ± 0.015	0.815 ± 0.011	0.765 ± 0.003	0.947 ± 0.001	0.960 ± 0.013	0.854 ± 0.039	0.947 ± 0.008	0.767 ± 0.017
ResNet-50	0.913 ± 0.004	0.842 ± 0.014	0.759 ± 0.002	**0.948** **±** **0.001**	0.949 ± 0.010	0.875 ± 0.032	0.957 ± 0.002	**0.776** ± **0.012**
DenseNet121	0.929 ± 0.020	0.669 ± 0.042	**0.773** ± **0.002**	**0.948** ± **0.001**	0.956 ± 0.007	**0.876** ± **0.015**	0.954 ± 0.004	0.762 ± 0.009
ConvNeXt	0.885 ± 0.008	0.471 ± 0.022	0.693 ± 0.001	0.887 ± 0.006	0.953 ± 0.003	0.840 ± 0.015	0.686 ± 0.019	0.542 ± 0.030
DINOv2-vits	0.976 ± 0.001	0.821 ± 0.003	0.560 ± 0.008	0.947 ± 0.001	0.896 ± 0.002	0.803 ± 0.004	0.839 ± 0.019	0.496 ± 0.028
DINOv2-vitb	0.973 ± 0.004	0.801 ± 0.028	0.573 ± 0.007	0.947 ± 0.001	0.875 ± 0.013	0.774 ± 0.023	0.830 ± 0.007	0.479 ± 0.008
BEITv2	0.968 ± 0.005	0.767 ± 0.036	0.578 ± 0.004	0.947 ± 0.001	0.901 ± 0.002	0.840 ± 0.002	0.754 ± 0.008	0.425 ± 0.035
Swin-T	0.825 ± 0.031	0.725 ± 0.002	0.510 ± 0.001	0.947 ± 0.001	0.824 ± 0.025	0.739 ± 0.037	0.666 ± 0.001	0.350 ± 0.001
MedFuse (dinov2_vits14 + Resnet18)	**0.989** ± **0.002**	**0.901** ± **0.012**	0.693 ± 0.001	**0.948** ± **0.001**	**0.965** ± **0.012**	0.855 ± 0.054	**0.958** ± **0.003**	0.751 ± 0.015

Methods	BreastMNIST		BloodMNIST		TissueMNIST		DermaMNIST	
	AUC	ACC	AUC	ACC	AUC	ACC	AUC	ACC
ResNet-18	**0.878** ± **0.020**	0.827 ± 0.022	0.996 ± 0.001	0.932 ± 0.001	0.930 ± 0.002	0.674 ± 0.009	0.914 ± 0.004	0.730 ± 0.009
ResNet-50	0.846 ± 0.018	**0.829** ± **0.010**	0.994 ± 0.001	0.912 ± 0.006	0.930 ± 0.002	0.678 ± 0.002	0.910 ± 0.002	0.733 ± 0.004
DenseNet121	0.822 ± 0.012	0.778 ± 0.006	0.991 ± 0.003	0.852 ± 0.048	0.934 ± 0.001	0.685 ± 0.002	0.913 ± 0.002	0.737 ± 0.005
ConvNeXt	0.767 ± 0.007	0.774 ± 0.039	0.994 ± 0.001	0.928 ± 0.003	0.928 ± 0.003	0.674 ± 0.005	0.911 ± 0.003	0.735 ± 0.004
DINOv2-vits	0.711 ± 0.037	0.729 ± 0.019	0.991 ± 0.008	0.902 ± 0.050	0.807 ± 0.082	0.497 ± 0.100	0.829 ± 0.006	0.679 ± 0.005
DINOv2-vitb	0.695 ± 0.048	0.729 ± 0.024	0.952 ± 0.014	0.748 ± 0.037	0.719 ± 0.018	0.399 ± 0.023	0.810 ± 0.011	0.671 ± 0.001
BEITv2	0.753 ± 0.012	0.731 ± 0.006	0.969 ± 0.001	0.777 ± 0.001	0.760 ± 0.002	0.458 ± 0.003	0.898 ± 0.002	0.708 ± 0.005
Swin-T	0.573 ± 0.055	0.731 ± 0.001	0.938 ± 0.022	0.695 ± 0.075	0.625 ± 0.015	0.331 ± 0.010	0.712 ± 0.003	0.669 ± 0.001
MedFuse (dinov2_vits14 + Resnet18)	0.854 ± 0.022	0.803 ± 0.032	**0.997** ± **0.001**	**0.951** ± **0.005**	**0.935** ± **0.001**	**0.692** ± **0.002**	**0.919** ± **0.002**	**0.743** ± **0.003**

Methods	RetinaMNIST		OrganAMNIST		OrganCMNIST		OrganSMNIST	
	AUC	ACC	AUC	ACC	AUC	ACC	AUC	ACC
ResNet-18	0.710 ± 0.005	0.500 ± 0.005	0.991 ± 0.003	0.863 ± 0.023	0.988 ± 0.001	0.868 ± 0.007	0.962 ± 0.007	0.745 ± 0.005
ResNet-50	0.714 ± 0.017	0.515 ± 0.005	0.991 ± 0.003	0.884 ± 0.020	0.989 ± 0.002	0.882 ± 0.011	0.954 ± 0.015	0.739 ± 0.009
DenseNet121	0.720 ± 0.005	0.502 ± 0.027	0.979 ± 0.004	0.797 ± 0.031	0.920 ± 0.002	0.741 ± 0.005	0.956 ± 0.006	0.726 ± 0.011
ConvNeXt	0.719 ± 0.006	0.501 ± 0.004	0.993 ± 0.001	0.887 ± 0.006	0.980 ± 0.002	0.812 ± 0.025	0.955 ± 0.005	0.637 ± 0.014
DINOv2-vits	0.716 ± 0.011	0.498 ± 0.026	0.984 ± 0.001	0.843 ± 0.004	0.982 ± 0.001	0.825 ± 0.005	0.912 ± 0.061	0.581 ± 0.134
DINOv2-vitb	0.716 ± 0.012	0.508 ± 0.008	0.982 ± 0.002	0.828 ± 0.013	0.863 ± 0.003	0.403 ± 0.023	0.840 ± 0.008	0.405 ± 0.017
BEITv2	0.706 ± 0.001	0.515 ± 0.004	0.955 ± 0.002	0.678 ± 0.009	0.963 ± 0.002	0.713 ± 0.006	0.946 ± 0.003	0.633 ± 0.008
Swin-T	0.694 ± 0.035	0.479 ± 0.021	0.800 ± 0.001	0.356 ± 0.001	0.910 ± 0.012	0.527 ± 0.047	0.895 ± 0.013	0.477 ± 0.034
MedFuse (dinov2_vits14 + Resnet18)	**0.749** ± **0.009**	**0.538** ± **0.024**	**0.997** ± **0.001**	**0.942** ± **0.003**	**0.992** ± **0.001**	**0.898** ± **0.007**	**0.978** ± **0.001**	**0.791** ± **0.008**

The observed dataset-to-dataset variation is consistent with differences in imaging modality, sample size, class structure, and the spatial scale of discriminative patterns. Microscopy and pathology tasks may benefit from the CNN branch's sensitivity to texture and local morphology, whereas organ CT subsets can benefit from the additional global representations supplied by the VFM branch. Conversely, chest x-ray and some smaller datasets show smaller or inconsistent gains, suggesting that the complementarity between CNN and VFM representations is task dependent rather than universal. Accordingly, the results provide evidence that late CNN–VFM feature fusion can be beneficial across a broad range of MedMNISTV2 classification tasks.

#### 4.3.2 Impact of VFM scale on dual-stream feature fusion performance

To further investigate the influence of VFM scale on dual-stream feature fusion, we evaluated DINOv2 variants of different scales in combination with ResNet-based convolutional backbones within the MedFuse framework. The detailed results of different CNN–VFM combinations across multiple MedMNISTV2 subsets are summarized in Table 3. As shown in the table, increasing the scale of the VFM does not necessarily lead to improved classification performance. Despite its substantially larger model capacity, the DINOv2-ViT-L/14 configuration combined with ResNet-50 does not consistently achieve the best performance across the evaluated datasets. In contrast, the lighter combination of DINOv2-ViT-S/14 and ResNet-18 achieves optimal or competitive results on several subsets, including PathMNIST, RetinaMNIST, and OrganSMNIST. These results indicate that a relatively lightweight VFM can effectively complement the local feature representations extracted by the convolutional branch.

**Table 3:** Performance comparison of different scales of DINOv2 and resNet within the medFuse framework.

Methods	PathMNIST		ChestMNIST		PneumoniaMNIST		OCTMNIST	
	AUC	ACC	AUC	ACC	AUC	ACC	AUC	ACC
dinov2_vitl14 + Resnet50	0.977 ± 0.005	0.867 ± 0.013	0.688 ± 0.002	0.947 ± 0.002	0.961 ± 0.006	0.852 ± 0.034	**0.963** **±** **0.003**	**0.778** ± **0.012**
dinov2_vitb14 + Resnet50	0.988 ± 0.005	0.898 ± 0.005	**0.697** ± **0.003**	0.947 ± 0.001	0.964 ± 0.011	**0.859** ± **0.018**	0.957 ± 0.007	0.749 ± 0.010
dinov2_vits14 + Resnet50	0.988 ± 0.003	0.888 ± 0.010	0.695 ± 0.002	0.947 ± 0.001	0.961 ± 0.010	0.848 ± 0.022	0.964 ± 0.008	0.772 ± 0.011
dinov2_vits14 + Resnet18	**0.989** ± **0.002**	**0.901** ± **0.012**	0.693 ± 0.001	**0.948** ± **0.001**	**0.965** ± **0.012**	0.855 ± 0.054	0.958 ± 0.003	0.751 ± 0.015

Methods	BreastMNIST		BloodMNIST		TissueMNIST		DermaMNIST	
	AUC	ACC	AUC	ACC	AUC	ACC	AUC	ACC
dinov2_vitl14 + Resnet50	0.840 ± 0.029	0.833 ± 0.017	0.997 ± 0.001	0.940 ± 0.004	0.934 ± 0.001	0.688 ± 0.005	0.918 ± 0.005	0.739 ± 0.007
dinov2_vitb14 + Resnet50	0.854 ± 0.020	0.823 ± 0.020	0.997 ± 0.001	0.954 ± 0.005	**0.937** ± **0.001**	**0.699** ± **0.002**	**0.920** ± **0.004**	**0.747** ± **0.001**
dinov2_vits14 + Resnet50	**0.855** ± **0.027**	**0.838** ± **0.004**	**0.998** ± **0.001**	**0.955** ± **0.002**	0.936 ± 0.001	0.692 ± 0.002	0.917 ± 0.001	0.737 ± 0.006
dinov2_vits14 + Resnet18	0.854 ± 0.022	0.803 ± 0.032	0.997 ± 0.001	0.951 ± 0.005	0.935 ± 0.001	0.692 ± 0.002	0.919 ± 0.002	0.743 ± 0.003

Methods	RetinaMNIST		OrganAMNIST		OrganCMNIST		OrganSMNIST	
	AUC	ACC	AUC	ACC	AUC	ACC	AUC	ACC
dinov2_vitl14 + Resnet50	0.724 ± 0.007	0.518 ± 0.016	0.997 ± 0.001	0.935 ± 0.007	0.912 ± 0.005	0.889 ± 0.007	0.976 ± 0.001	0.783 ± 0.003
dinov2_vitb14 + Resnet50	0.742 ± 0.005	0.518 ± 0.018	0.997 ± 0.001	0.939 ± 0.005	0.991 ± 0.001	**0.906** ± **0.005**	0.976 ± 0.001	0.782 ± 0.002
dinov2_vits14 + Resnet50	0.731 ± 0.008	0.531 ± 0.020	**0.998** ± **0.001**	**0.944** ± **0.002**	**0.992** ± **0.001**	0.897 ± 0.004	0.976 ± 0.001	0.783 ± 0.009
dinov2_vits14 + Resnet18	**0.749** ± **0.009**	**0.538** ± **0.024**	0.997 ± 0.001	0.942 ± 0.003	**0.992** ± **0.001**	0.898 ± 0.007	**0.978** ± **0.001**	**0.791** ± **0.008**

To further explore the correlation between model scale and overall performance, this study visualized the associations between the parameter size of different model combinations and their average AUC and ACC values, as presented in Figure 3 The visualization reveals no distinct positive correlation between modal parameter quantity and average performance. The largest model combination, dinov2_vitl14 paired with ResNet50, fails to deliver the best average performance, whereas-scale pairing of dinov2_vits14 and ResNet18 shows prominent competitiveness. The results show that the performance of medical image classification models does not depend entirely on the size of VFMs. Instead, it is largely decided by how well global semantic features from Vision Transformers pair with fine-grained local features extracted by CNNs

**Figure 3:**
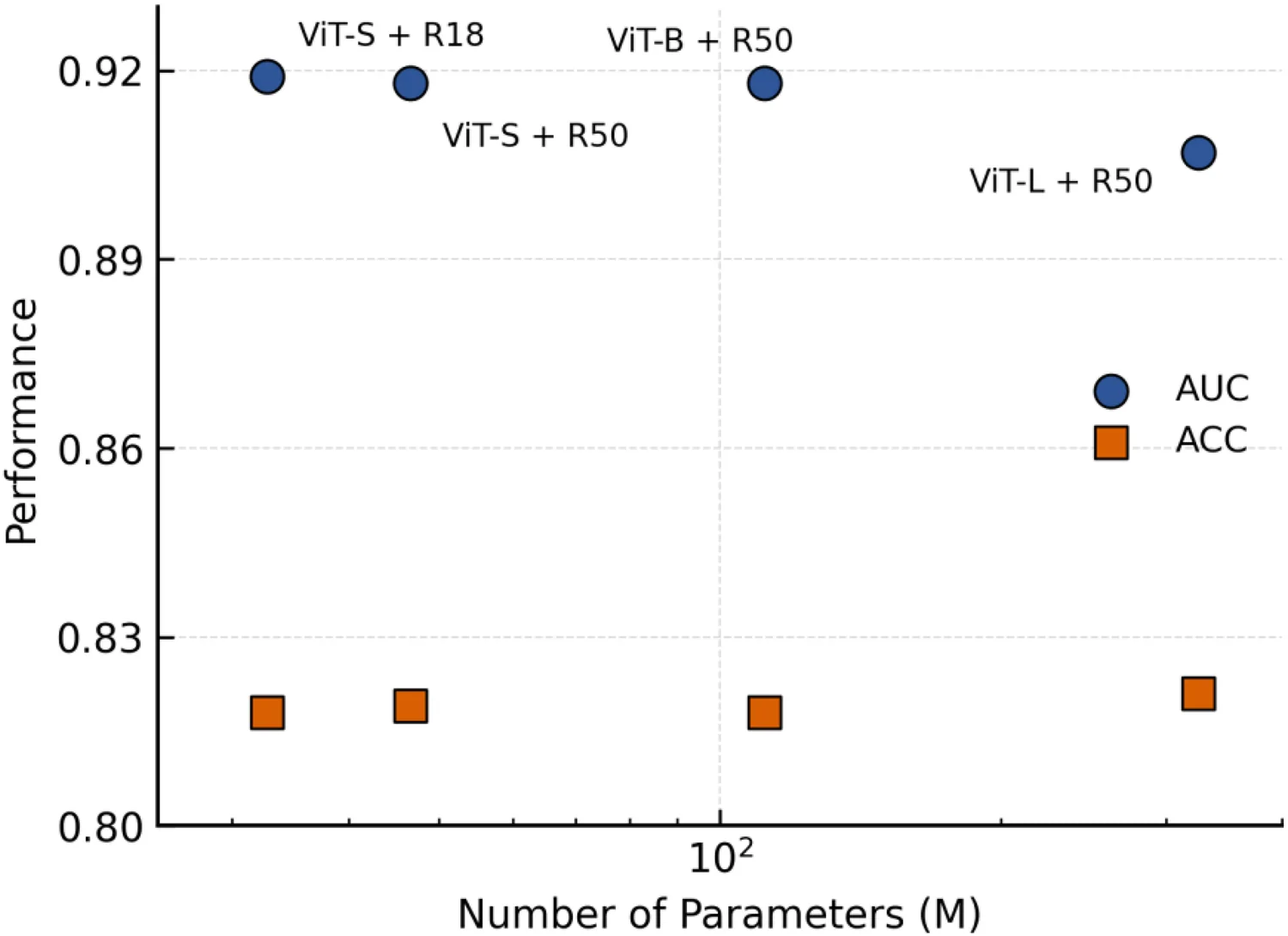
Relationship between parameter count and average AUC/ACC performance for different DINOv2–resNet combinations.

To sum up, large VFMs cannot bring their full strengths into play for medical imaging tasks. Limited labeled medical data, domain gaps, and redundant features all hold them back. By contrast, when combined with CNNs within our dual-stream fusion structure, small and medium-sized VFMs deliver more stable and efficient results

To further evaluate the computational efficiency of MedFuse, we analyzed the computational complexity of the default DINOv2-ViT-S/14 + ResNet-18 configuration with an input resolution of 224 × 224 pixels. The complete model contains approximately 34 M parameters, of which approximately 12 M are trainable because the pretrained VFM backbone remains frozen during MedFuse training. The computational complexity is approximately 6.5 GFLOPs per image. The model requires approximately 7.2 GB of peak GPU memory with a batch size of 32. The average inference time is approximately 8.4 ms per image, corresponding to a throughput of approximately 119 images per second. These results indicate that freezing the pretrained VFM substantially reduces the number of trainable parameters, although the complete VFM still participates in the forward pass and therefore contributes to the overall computational and memory costs. Consequently, MedFuse introduces additional inference overhead compared with a single-backbone CNN, representing a trade-off between complementary feature representation and computational efficiency. Figure 3.

#### 4.3.3 Performance of different CNN and VFM backbone combinations within the medFuse framework

We also evaluated different CNN and VFM backbone pairs to examine the robustness of the late-fusion framework to architectural choice. The combinations include ConvNeXt-Tiny paired with DINOv2, DenseNet-121 with DINOv2, ResNet-50 with BEiTv2, and ResNet-50 with Swin-T. Detailed results are summarized in Table 4. The results show that performance varies across datasets and backbone pairs, indicating that MedFuse is not restricted to a single CNN or VFM family but that the benefit of fusion is task- and pairing-dependent. Accordingly, these experiments support architectural flexibility of the framework rather than universal improvement for every backbone combination and dataset.

**Table 4:** Classification performance comparison of different CNN and VFM combinations within the medFuse framework.

Methods	PathMNIST		ChestMNIST		PneumoniaMNIST		OCTMNIST	
	AUC	ACC	AUC	ACC	AUC	ACC	AUC	ACC
ConvNeXt-Tiny + DINOv2-ViT-B/14	**0.987** **±** **0.002**	0.895 ± 0.004	0.615 ± 0.002	**0.947** ± **0.001**	0.960 ± 0.003	0.861 ± 0.013	0.959 ± 0.001	0.776 ± 0.003
DenseNet-121 + DINOv2-ViT-B/14	0.979 ± 0.004	0.890 ± 0.017	**0.706** ± **0.001**	0.945 ± 0.001	0.969 ± 0.008	**0.894** ± **0.007**	**0.969** ± **0.006**	**0.792** ± **0.013**
ResNet50 + BEITv2	0.985 ± 0.004	0.875 ± 0.009	0.688 ± 0.001	**0.947** ± **0.001**	0.957 ± 0.004	0.878 ± 0.008	0.963 ± 0.001	0.791 ± 0.004
ResNet50 + Swin-T	0.984 ± 0.006	**0.909** ± **0.007**	0.697 ± 0.002	0.944 ± 0.003	**0.967** ± **0.006**	0.885 ± 0.009	0.961 ± 0.003	0.780 ± 0.012

Methods	BreastMNIST		BloodMNIST		TissueMNIST		DermaMNIST	
	AUC	ACC	AUC	ACC	AUC	ACC	AUC	ACC
ConvNeXt-Tiny + DINOv2-ViT-B/14	0.711 ± 0.001	0.741 ± 0.021	0.995 ± 0.001	0.935 ± 0.002	0.934 ± 0.004	0.686 ± 0.002	0.914 ± 0.001	0.737 ± 0.004
DenseNet-121 + DINOv2-ViT-B/14	**0.839** ± **0.009**	0.811 ± 0.011	**0.998** ± **0.001**	**0.961** ± **0.002**	0.941 ± 0.001	**0.706** ± **0.002**	**0.916** ± **0.001**	**0.747** ± **0.001**
ResNet50 + BEITv2	0.834 ± 0.001	**0.840** ± **0.001**	0.994 ± 0.001	0.933 ± 0.001	**0.937** ± **0.001**	0.701 ± 0.002	**0.916** ± **0.003**	0.744 ± 0.005
ResNet50 + Swin-T	0.833 ± 0.010	0.810 ± 0.022	**0.998** ± **0.001**	0.942 ± 0.002	0.936 ± 0.001	0.697 ± 0.002	0.915 ± 0.002	0.738 ± 0.007

Methods	RetinaMNIST		OrganAMNIST		OrganCMNIST		OrganSMNIST	
	AUC	ACC	AUC	ACC	AUC	ACC	AUC	ACC
ConvNeXt-Tiny + DINOv2-ViT-B/14	0.722 ± 0.005	0.518 ± 0.006	0.996 ± 0.001	0.925 ± 0.005	0.992 ± 0.001	0.895 ± 0.001	0.973 ± 0.002	0.764 ± 0.012
DenseNet-121 + DINOv2-ViT-B/14	0.721 ± 0.004	**0.534** ± **0.004**	**0.998** ± **0.001**	**0.945** ± **0.004**	**0.993** ± **0.001**	0.906 ± 0.005	**0.979** ± **0.001**	**0.794** ± **0.006**
ResNet50 + BEITv2	**0.729** ± **0.006**	0.528 ± 0.009	0.997 ± 0.001	0.935 ± 0.005	0.992 ± 0.001	0.902 ± 0.005	0.976 ± 0.001	0.786 ± 0.005
ResNet50 + Swin-T	0.722 ± 0.003	0.515 ± 0.004	**0.998** ± **0.001**	0.937 ± 0.006	0.992 ± 0.001	**0.908** ± **0.001**	0.974 ± 0.001	0.772 ± 0.012

To summarize performance changes relative to the ResNet-50 baseline, this study calculated average AUC and ACC differences across ten sub-datasets, excluding ChestMNIST and BreastMNIST, as shown in Figure 4. On this selected set of tasks, the evaluated CNN-VFM pairs show positive average changes relative to ResNet-50, with DenseNet-121 combined with DINOv2 showing the largest average increase among the tested combinations. These aggregate results should not be interpreted as evidence that fusion improves every individual dataset or that one pairing is universally optimal. Rather, they indicate that the simple late-fusion framework can accommodate multiple heterogeneous backbones, while the magnitude of benefit depends on the specific representation pair and task.

**Figure 4:**
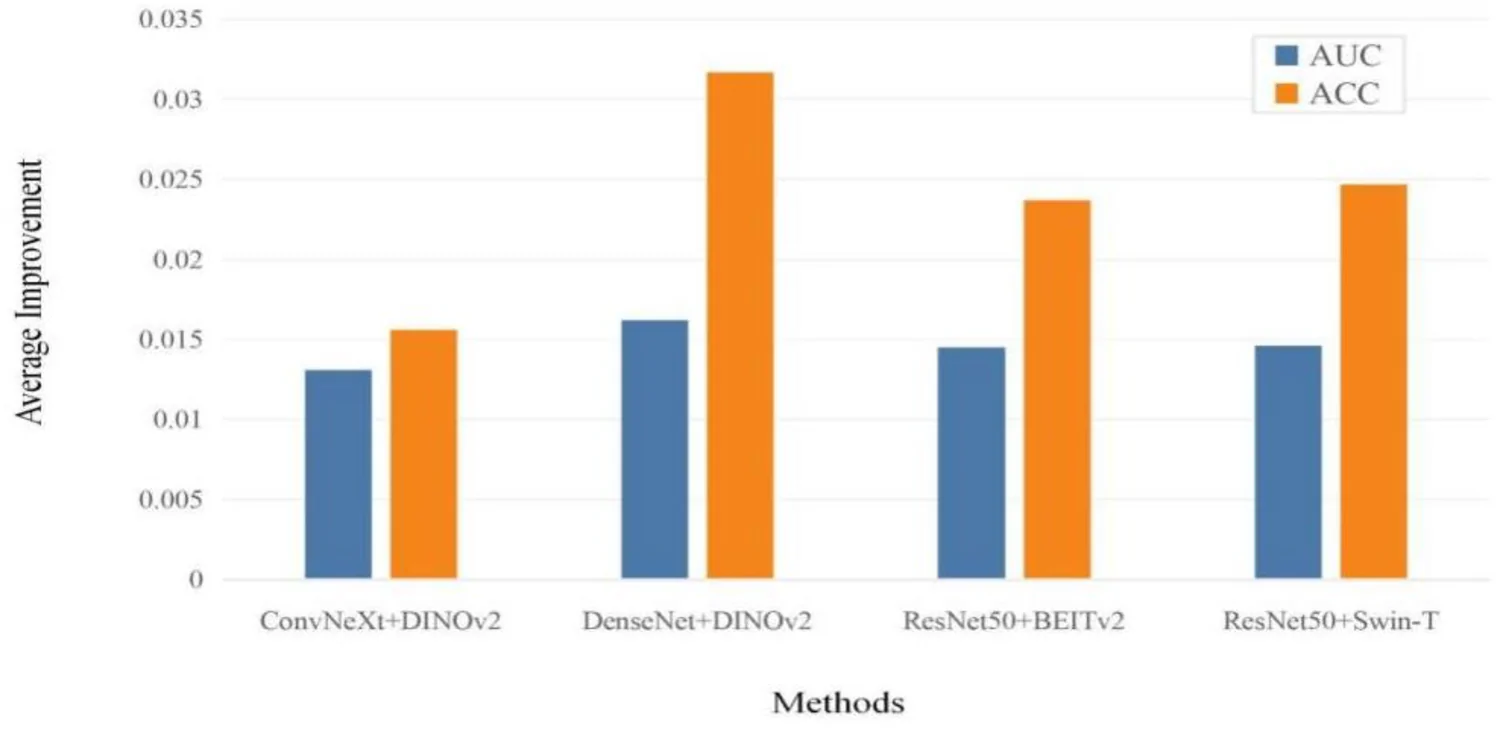
Average performance improvement of different CNN–VFM combinations relative to the ResNet50 baseline.

#### 4.3.4 Impact of different feature fusion strategies on model performance

To explore the influence of fusion strategies on MedFuse performance model, this study compared three fusion schemes embedded in the dual-stream architecture: concatenation (Concat), adaptive weight fusion (Weight), and attention-based fusion (Attention). The Concat strategy directly merges global semantic and local texture features. Weight fusion dynamically balances the contribution of two feature streams via trainable adaptive coefficients. Attention fusion leverages attention mechanisms to capture intrinsic correlations among diverse features. Table 5 presents detailed results of Weight and Attention fusion. Both strategies deliver valid improvements on partial datasets. Weight fusion reaches AUC values of 0.997, 0.997, and 0.975 on BloodMNIST, OrganAMNIST, and OrganSMNIST, respectively, proving adaptive weighting can effectively integrate complementary features extracted by CNN and VFM branches. Attention fusion obtains competitive AUC scores of 0.705 and 0.992 on ChestMNIST and OrganCMNIST, which helps the model concentrate on task-discriminative features. Nevertheless, the two advanced fusion methods show unstable overall performance. In particular, attention fusion suffers obvious accuracy declines on PathMNIST, BreastMNIST, OrganAMNIST, and OrganSMNIST. it reveals that sophisticated attention fusion cannot steadily bring reliable performance for medical image classification tasks.

**Table 5:** Performance comparison of weight and attention fusion strategies within the medFuse framework.

Methods	PathMNIST		ChestMNIST		PneumoniaMNIST		OCTMNIST	
	AUC	ACC	AUC	ACC	AUC	ACC	AUC	ACC
Weight	0.986 ± 0.002	0.890 ± 0.003	0.693 ± 0.004	0.944 ± 0.001	0.970 ± 0.008	0.891 ± 0.004	0.959 ± 0.001	0.795 ± 0.009
Attention	0.942 ± 0.008	0.748 ± 0.012	0.705 ± 0.004	0.945 ± 0.002	0.963 ± 0.006	0.898 ± 0.026	0.961 ± 0.001	0.746 ± 0.001

Methods	BreastMNIST		BloodMNIST		TissueMNIST		DermaMNIST	
	AUC	ACC	AUC	ACC	AUC	ACC	AUC	ACC
Weight	0.849 ± 0.022	0.83 ± 0.012	0.997 ± 0.001	0.947 ± 0.003	0.938 ± 0.001	0.689 ± 0.016	0.914 ± 0.004	0.736 ± 0.010
Attention	0.731 ± 0.011	0.763 ± 0.016	0.992 ± 0.001	0.912 ± 0.001	0.937 ± 0.002	0.694 ± 0.003	0.913 ± 0.003	0.740 ± 0.007

Methods	RetinaMNIST		OrganAMNIST		OrganCMNIST		OrganSMNIST	
	AUC	ACC	AUC	ACC	AUC	ACC	AUC	ACC
Weight	0.733 ± 0.007	0.524 ± 0.005	0.997 ± 0.001	0.937 ± 0.005	0.991 ± 0.001	0.900 ± 0.007	0.975 ± 0.002	0.762 ± 0.006
Attention	0.727 ± 0.003	0.539 ± 0.010	0.964 ± 0.001	0.728 ± 0.005	0.992 ± 0.001	0.903 ± 0.001	0.946 ± 0.001	0.648 ± 0.004

Using the DINOv2-ViT-S/14 + ResNet-18 configuration as the Concat reference, Figure 5 compares the performance differences relative to Weight and Attention fusion. Concat is often favorable across the evaluated subsets. Its main advantage is information preservation: both pooled branch representations are retained without projection or pre-filtering, and the fusion operator itself introduces no learned parameters. This reduces fusion-layer complexity and avoids additional optimization instability on small datasets. The trade-off is that concatenation increases the dimensionality of the classifier input and does not explicitly model cross-branch interactions. Weight fusion has lower representational flexibility because it compresses the two streams through learned coefficients, whereas attention can model interactions but introduces additional parameters and optimization sensitivity. The results therefore support Concat as a strong simple baseline for this benchmark rather than establishing it as universally superior to learned fusion mechanisms.

**Figure 5:**
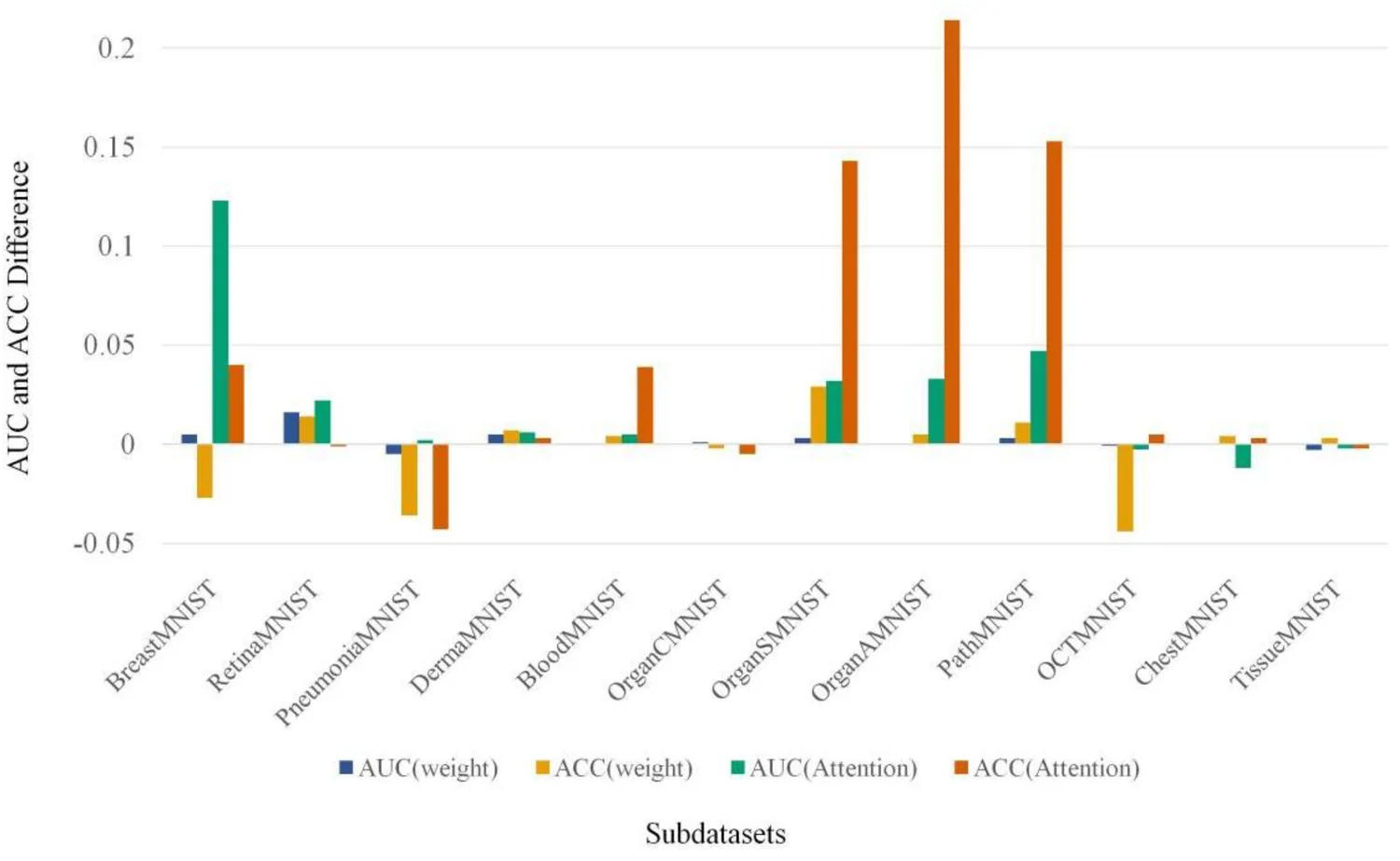
Comparison of AUC/ACC differences between concat fusion and weight, attention fusion strategies across Sub-datasets.

#### 4.3.5 Qualitative analysis of representative predictions and activation maps

To provide a more intuitive understanding of the classification behavior of MedFuse, we further conducted a qualitative analysis using test samples from BloodMNIST, BreastMNIST, DermaMNIST, and OrganCMNIST. These datasets cover substantially different medical imaging modalities and visual characteristics, including blood-cell microscopy, breast ultrasound, dermoscopy, and CT-derived organ images. As shown in Table 6, for each dataset, one correctly classified example and one misclassified example are presented together with the raw image, ground-truth class, predicted class, prediction confidence, and corresponding Grad-CAM++ heatmap.

**Table 6:** Qualitative analysis of representative correct and incorrect medFuse predictions with grad-CAM++ visualizations.

Dataset	Raw Image	Ground Truth Class	Predicted Class	Correct?	Confidence (%)	Grad-CAM++ Heatmap
(a) BloodMNIST (8 classes)	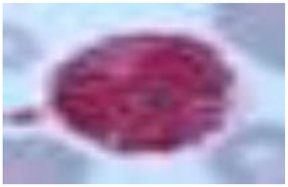	Basophil	Basophil	✓	94.8	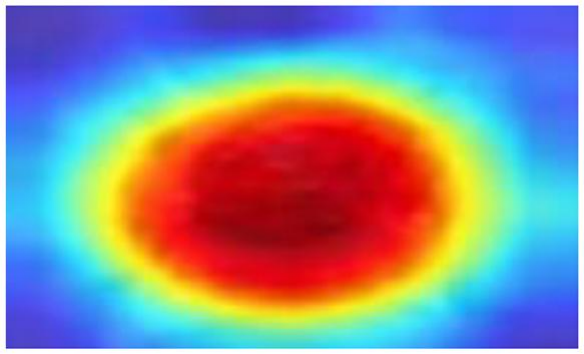
	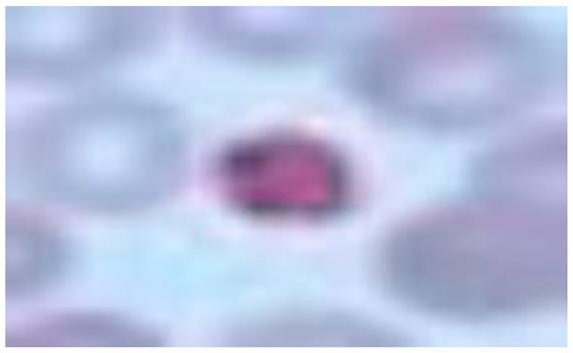	Erythroblast	Lymphocyte	✕	57.3	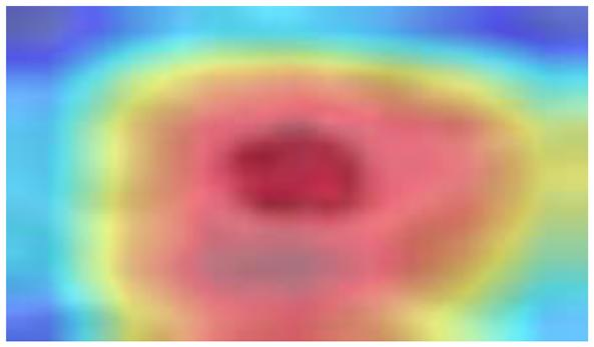
(b)Breast MNIST (2 classes)	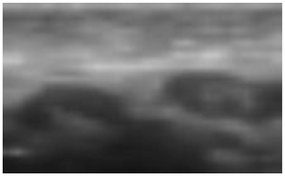	Malignant	Malignant	✓	85.6	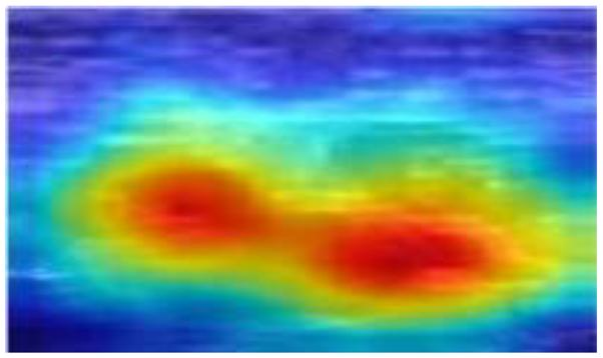
	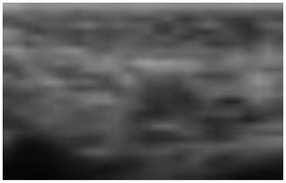	Malignant	Normal/Benign	✕	56.7	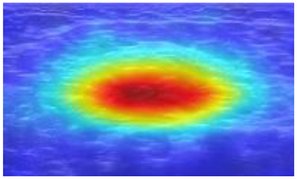
(c)DermaMNIST (7 classes)	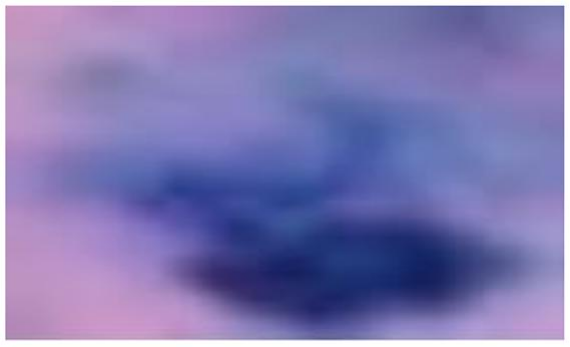	Melanoma	Melanoma	✓	87.6	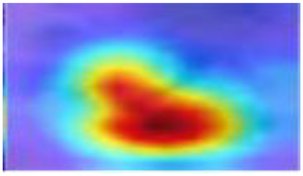
	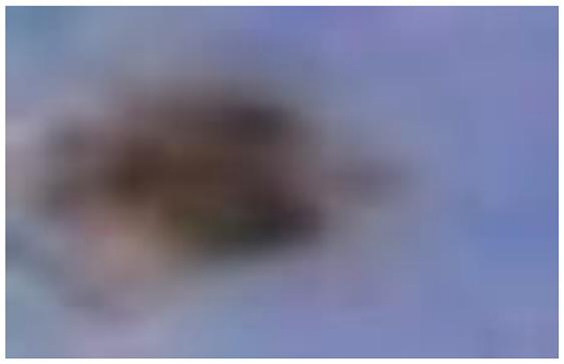	Melanocytic nevi	Melanoma	✕	62.1	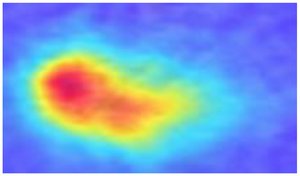
(d)OrganCMNIST (11 classes)	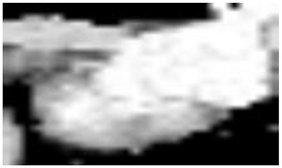	Heart	Heart	✓	91.3	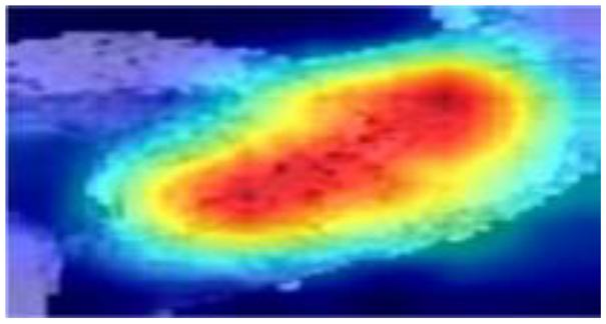
	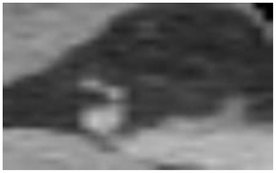	Liver	Spleen	✕	61.4	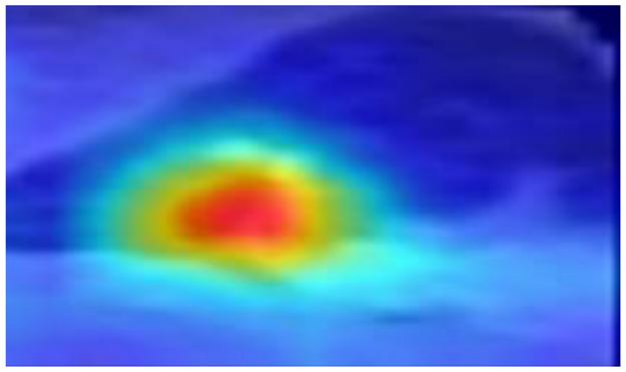

For the correctly classified examples, MedFuse generally produces relatively high prediction confidence, ranging from 85.6% to 94.8%. In BloodMNIST, the Basophil sample is correctly classified with a confidence of 94.8%, and the strongest Grad-CAM++ response is concentrated around the cellular region. Similarly, the correctly classified malignant BreastMNIST sample achieves a confidence of 85.6%, with the activation primarily distributed over the structurally salient region in the ultrasound image. For DermaMNIST, the melanoma sample is correctly recognized with 87.6% confidence, and the high-response region overlaps substantially with the lesion area. In OrganCMNIST, the heart sample is correctly classified with 91.3% confidence, while the activation map broadly follows the prominent anatomical structure in the CT-derived image. In these representative examples, successful predictions are accompanied by relatively concentrated responses over visually relevant structures rather than unrelated background regions.

The misclassified examples in Table 6 provide additional insight into potential failure modes. The BloodMNIST Erythroblast sample is incorrectly predicted as Lymphocyte with a confidence of 57.3%, suggesting uncertainty between morphologically similar cellular patterns. In BreastMNIST, a malignant sample is misclassified as Normal/Benign with 56.7% confidence. The relatively moderate confidence and dispersed activation suggest that the visual evidence available to the model is less discriminative than in the correctly classified example. For DermaMNIST, a Melanocytic nevi sample is predicted as Melanoma with 62.1% confidence. The Grad-CAM++ response still overlaps with the lesion region, indicating that the error may arise not from attention to irrelevant background but from difficulty in distinguishing visually similar lesion characteristics. Likewise, the OrganCMNIST liver sample is incorrectly classified as spleen with 61.4% confidence, while the activation remains concentrated on an anatomically relevant region.

These results indicate that the selected correctly classified examples exhibit relatively high confidence and concentrated responses over task-relevant structures, whereas the selected failure cases show moderate confidence and less discriminative or partially ambiguous activation patterns. Importantly, the misclassified examples demonstrate that localization of a visually relevant region does not necessarily guarantee correct classification, particularly when different categories share similar morphological, textural, or anatomical characteristics.

## 5 Discussion

This study systematically investigates a simple dual-stream framework that combines task-adaptive local representations from a trainable CNN with global representations extracted from a frozen pretrained VFM. Rather than introducing a new mechanism-level fusion module, MedFuse is designed as a flexible benchmark framework for examining whether heterogeneous CNN and VFM representations can provide complementary information for medical image classification. By keeping the VFM backbone frozen while optimizing the CNN branch and the subsequent fusion and classification layers, the framework preserves the transferable representations of the pretrained VFM while allowing the convolutional stream to adapt to task-specific medical image characteristics. This asymmetric training strategy also reduces the number of trainable parameters and optimization cost compared with full fine-tuning of both branches.

Across the 12 two-dimensional MedMNISTV2 subsets, MedFuse demonstrates competitive performance against single-backbone CNN and Transformer/VFM baselines on many classification tasks, although the magnitude of improvement varies substantially across datasets. This task-dependent behavior may be associated with differences in imaging modality, dataset size, class distribution, anatomical structure, and the spatial scale of discriminative patterns. For example, microscopy and histopathology images contain rich texture and fine-grained morphological patterns for which CNN-based local representations can be particularly informative. In contrast, organ-related CT subsets contain broader anatomical structures and spatial relationships, where the global contextual representations extracted by the VFM may provide additional complementary information. Chest x-ray and some relatively small datasets exhibit smaller or less consistent improvements, indicating that the complementarity between CNN and VFM representations is task dependent rather than universal.

Experiments using different CNN–VFM combinations further demonstrate that the behavior of MedFuse is not restricted to a single backbone architecture. The CNN branch covers representative residual, densely connected, and modern convolutional architectures, while the VFM branch includes DINOv2 variants at different scales as well as alternative Transformer architectures such as BEiTv2 and Swin-T. The results indicate that increasing VFM model size does not consistently lead to better classification performance. Instead, the compatibility between the local representations extracted by the CNN and the global representations provided by the VFM appears to play an important role. This observation suggests that selecting complementary backbone representations may be more important than simply increasing model capacity.

The comparison of fusion strategies also provides useful empirical observations. Late concatenation directly preserves the pooled feature coordinates from both branches and introduces essentially no additional learnable parameters in the fusion operation itself. It therefore provides a simple way to retain both CNN and VFM representations without applying feature filtering or compression before classification. In comparison, adaptive weighting and attention-based fusion introduce additional feature-selection or interaction mechanisms and require additional parameterization and optimization. The experimental results show that these more complex strategies do not consistently outperform direct concatenation across the evaluated datasets. Nevertheless, concatenation also has clear limitations. In particular, it increases the dimensionality of the fused representation and consequently enlarges the input dimension of the subsequent classification head. Moreover, concatenation does not explicitly model interactions between the CNN and VFM representations; instead, such cross-branch relationships must be learned implicitly by the downstream classifier. Therefore, the results should not be interpreted as demonstrating that concatenation is universally superior to more sophisticated fusion mechanisms, but rather that it provides a competitive and stable trade-off between information preservation and fusion complexity in the present benchmark.

All experiments were independently repeated three times, and the quantitative results are reported as the mean ± standard deviation across the three runs. This repeated-evaluation protocol provides an estimate of experimental variability and was applied consistently across the evaluated configurations. Nevertheless, some performance differences between MedFuse and competing methods remain relatively modest. Since formal statistical significance tests were not performed in the present study, small differences should be interpreted with appropriate caution and should not be regarded as statistically significant solely on the basis of the reported mean values.

Several limitations of the present study should therefore be acknowledged. First, although the evaluation covers all 12 two-dimensional MedMNISTV2 subsets and therefore represents a broad range of standardized biomedical image classification tasks, all experiments rely on the benchmark's predefined internal train, validation, and test splits. The current results consequently support robustness across different tasks within MedMNISTV2 but do not establish clinical generalization to independent institutions, patient populations, imaging devices, or acquisition protocols. In future work, we plan to evaluate MedFuse on larger, independent, and multi-center medical imaging datasets with compatible imaging characteristics and label definitions to further investigate its cross-dataset robustness and clinical generalizability.

Second, although three independent runs were conducted for each experiment and the results are reported as mean ± standard deviation, the present study does not include stratified k-fold cross-validation or 95% confidence intervals. More extensive repeated evaluations, confidence-interval analysis, and stratified k-fold cross-validation will be considered in future work to provide more robust estimates of model performance and variability. Formal statistical significance analyses will also be incorporated where appropriate to determine whether relatively small differences between competing approaches represent statistically reliable improvements.

Third, the present study primarily adopts a frozen-VFM and trainable-CNN strategy. Additional training-strategy ablations, including full fine-tuning of the VFM together with the CNN, freezing both feature extractors while training only the fusion/classification head, and parameter-efficient adaptation of the VFM, would provide a more comprehensive understanding of the influence of optimization strategies. These settings will be systematically investigated in future work, particularly with respect to the trade-off among classification performance, computational cost, and trainable parameter efficiency.

Overall, the present findings suggest that the benefit of CNN–VFM fusion depends not only on model capacity but also on dataset characteristics, backbone compatibility, training strategy, and the manner in which heterogeneous representations are integrated. MedFuse therefore provides a simple and flexible framework for systematically studying these factors across diverse medical image classification tasks.

## 6 Conclusion

This study presents MedFuse, a simple and flexible dual-stream framework for medical image classification that combines task-adaptive local representations from a trainable CNN with global representations extracted from a frozen pretrained VFM through late feature concatenation. The primary contribution of the study lies not in proposing a new mechanism-level fusion operator, but in systematically evaluating the complementarity of CNN and VFM representations across a broad standardized medical image classification benchmark.

Experiments across all 12 two-dimensional MedMNISTV2 subsets demonstrate that MedFuse is competitive with single-backbone models on a wide range of tasks. Evaluations involving different CNN–VFM combinations and different VFM scales further show that larger models do not necessarily provide better performance and that the compatibility between heterogeneous representations plays an important role. The comparison of concatenation, adaptive weighting, and attention-based fusion strategies also indicates that simple late concatenation can provide a competitive balance among feature preservation, fusion complexity, and performance stability across diverse tasks.

Taken together, these results support the practical value of combining convolutional local representations with pretrained global representations for medical image classification, while also showing that the resulting benefits are task dependent rather than universal. Future work will extend MedFuse to independent and multi-center clinical datasets, incorporate more comprehensive statistical evaluation and training-strategy ablations, and further investigate efficient and interpretable mechanisms for modeling interactions between CNN and VFM representations.

## Data Availability

The original contributions presented in the study are included in the article/Supplementary Material, further inquiries can be directed to the corresponding author/s.
